# Effect of the Implementation of a Structured Diet Management Plan on the Severity of Obstructive Sleep Apnea: A Systematic Review

**DOI:** 10.1007/s13668-022-00445-w

**Published:** 2022-11-26

**Authors:** Aikaterini Rokou, Anna Eleftheriou, Christina Tsigalou, Ioulianos Apessos, Evangelia Nena, Maria Dalamaga, Athanasios Voulgaris, Paschalis Steiropoulos

**Affiliations:** 1 Department of Pneumonology, Medical School, Democritus University of Thrace, Alexandroupolis, Greece; 2 Laboratory of Microbiology, Medical School, Democritus University of Thrace, Alexandroupolis, Greece; 3 MSc Program in Sleep Medicine, Medical School, Democritus University of Thrace, Dragana, 68100 Alexandroupolis Greece; 4 Laboratory of Social Medicine, Medical School, Democritus University of Thrace, Alexandroupolis, Greece; 5 Department of Biological Chemistry, Medical School, National and Kapodistrian University of Athens, Athens, Greece

**Keywords:** Obstructive sleep apnea, Apnea-hypopnea index, Weight loss diet, Mediterranean diet, Continuous positive airway pressure

## Abstract

***Purpose of Review*:**

The prevalence of obstructive sleep apnea (OSA) is increasing worldwide, in line with the increase in obesity prevalence. Taken into consideration the low compliance rates to continuous positive airway pressure (CPAP) treatment, and since obesity is a risk factor for OSA, these patients should receive additional counseling for weight loss through a diet plan. The aim of this review is to examine the role of a structured diet management plan on OSA severity, nocturnal oxygen indices, and subjective sleep parameters.

***Recent Findings*:**

Τhis systematic review of the literature resulted in four studies and demonstrated that severity of OSA, assessed by the apnea-hypopnea index, is reduced by a dietary management plan when delivered through an educational program. Moreover, nocturnal oxygenation is improved, as well as subjective sleep parameters, when initiating a diet on top of CPAP use.

***Summary*:**

In summary, the present systematic review reports on the beneficial effects of a structured diet management plan in patients with OSA. Although CPAP remains the gold standard of OSA treatment, a specific dietary plan should be sought when managing patients with OSA. Nevertheless, still the evidence is low, and further research on this field is needed to reduce the burden of OSA.

## Introduction

Obstructive sleep apnea (OSA) is the most prevalent form of sleep-disordered breathing defined by recurrent episodes of complete (apneas) or partial (hypopneas) upper airway obstruction during sleep [1]. These episodes often lead to oxygen desaturation, sleep fragmentation, heart rate variability, and nighttime and daytime symptoms, i.e., snoring and excessive daytime sleepiness [2–4]. OSA shows an increasing prevalence, affecting approximately 1 billion adults aged 30–69 years globally [5••]. Established risk factors for OSA are male gender, age over 60 years, obesity, smoking, and family history [6]. In addition, commonly reported comorbidities of OSA include cardiovascular disease, hypertension, cognitive impairment, and diabetes which are often aggravated by OSA [7].

The first-line treatment for OSA is the application of continuous positive airway pressure (CPAP), which splints the upper airway open and prevents the presence of disordered breathing when the patient sleeps [8••]. Despite its effectiveness, this type of treatment is often not well-tolerated by many patients, and thus, compliance with CPAP is not guaranteed and often challenging when managing patients with OSA [9, 10••]. Specifically, recent reports claim that up to 50% of patients with OSA refuse to continue their CPAP treatment during the first year, and the level of non-adherence to CPAP falls even more over the years [11••, 12]. Alternatively, mandibular advancement devices may be used to reposition properly the tongue or the mandible and avoid breathing pauses during sleep [13••]. However, their use is limited, mainly because they apply in selected patients who fail to tolerate CPAP or in those with mild-moderate OSA [13••]. Other non-conservative measures, such as hypoglossal nerve stimulator or upper airway surgery, may be considered in even fewer patients who fulfill specific requirements, as per guidelines of OSA treatment [13••].

Since obesity is a risk factor for OSA, one should consider, among other measures, a dedicated dietary management program aimed at reducing the excess body weight. Studies have shown that weight reduction of even 5–10% may lead to the improvement of OSA-related symptoms [14, 15]. Weight loss can be achieved either by bariatric surgery, lifestyle interventions, or anti-obesity medications [16]. Both bariatric surgery and drugs have been proven to be effective in obese patients suffering from OSA by reducing the AHI and thus the severity of OSA [13••, 17–19]. Nevertheless, they remain an invasive form of treatment, and especially, bariatric surgery carries possible complications along with anxiety, pain, and dietary restrictions [20]. Lifestyle modifications, such as diet and exercise, remain an easy, non-invasive alternative to achieve weight loss [20]. Unfortunately, while weight loss can lead to reduction of OSA severity and improvement of OSA-related symptoms, it seldom stands as the only treatment, and many patients with OSA will require further treatment modalities such as CPAP [13••].

Ever-growing evidence suggests that weight loss through lifestyle interventions has been efficient in ameliorating the severity of symptoms in patients with OSA [21, 22]. The importance of weight management strategies has also been underlined by the American Thoracic Society official clinical practice guidelines for the management of patients with OSA [17]. A systematic review and meta-analysis showed that calorie restrictive diets as part of intensive lifestyle programs can reduce the apnea-hypopnea index (AHI) [23], i.e., the average number of apneas and hypopneas per hour of sleep that represents the main metric of OSA severity [8••]. A more recent meta-analysis, of 13 randomized controlled trials and 22 uncontrolled before-and-after studies [24], showed a beneficial effect of diet-induced weight loss on OSA parameters, such as AHI, oxygen desaturation index (ODI), arousal index, and excessive daytime sleepiness (EDS). However, novel data have recently emerged [25••, 26••], while no systematic review has synthesized the evidence of a structured dietary program on OSA severity since then. Therefore, the aim of the study is to evaluate and summarize the existing evidence regarding the effect of different types of dietary management plans on OSA severity and symptoms.

## Methods

### Study Protocol

#### Information Sources and Search Strategy

A systematic review of the relevant publications was conducted using Medline/PubMed, Scopus, and the Cochrane Library from study inception to March 2022. Additionally, reference lists were checked, and publications not included in the abovementioned databases were added. The keywords used in our search were (“sleep disordered breathing, sleep related breathing disorders, sleep apnea, sleep apnoea, OSA, obstructive sleep apnea” or “obstructive sleep apnoea”) AND (“diet” or “diet patterns” or “dietary patterns” or “Mediterranean diet” or “protein” or “carbohydrates” or “vegan” or “low-carb” or “paleo” or “raw food” or “vegetarian” or “low-fat” or “high-protein” or “meals” or “fruits” or “gluten-free”). The search strategy was confined to studies published in English language.

#### Study Selection and Data Management

Eligible studies for inclusion complied with certain criteria. Specifically, included were prospective studies reporting on a certain diet plan, not aimed at calorie restriction, and its effect on the severity of OSA and/or symptoms related to OSA; objectively diagnosed OSA by a sleep study (either polysomnography or a home sleep apnea testing); sleep scoring according to the updated rules for respiratory event scoring of the American Academy of Sleep Medicine (AASM) in 2012; and adult patients.

Randomized controlled trials (RCTs); cross-sectional, cohort, interventional, and observational follow-up studies; and longitudinal studies were deemed eligible for inclusion. Both prospective and retrospective studies were included. Case reports, letters to the editor, systematic reviews, case series, and books were excluded.

Two reviewers (AE, AR) screened independently the titles and abstracts of the retrieved articles based on the abovementioned inclusion and exclusion criteria. After the initial screening, a full-text assessment of the articles was performed. Disagreements were settled through discussion, but in the case of inconclusive decision, a third reviewer (AV) resolved the conflict.

Afterwards, data extraction of the included articles was performed in a custom data extraction sheet, which presented the following information: first author/year, study design, study population, study groups, intervention, outcomes/associations, follow-up period, and results.

### Definitions, Interventions, and Outcome Measures

#### Definitions of Study Measurements

Obstructive sleep apnea was defined as the presence of either at least 15 obstructive respiratory episodes (apneas, hypopneas, or respiratory effort-related arousals) per hour of recorded sleep or at least 5 episodes in line with symptoms such as daytime sleepiness, loud snoring, observed breathing disruptions, or awakenings due to gasping or choking or associated comorbidities [27]. Sleep and respiratory events were scored according to 2012 rules of the AASM [28]. Regarding diet definition, the following types of diets were included: low-salt diet, Mediterranean diet, prudent diet, and low-energy high-protein diet (Table 1). The primary outcome of the study was the effect of a dietary intervention program on severity of OSA as assessed by the AHI. Secondary outcome measures were the effect of a dietary intervention program on average and minimum SpO_2_, the time spent with SpO_2_ below 90%, and the oxygen desaturation index as well as on the subjective sleep parameters (i.e., excessive daytime sleepiness, insomnia, sleep quality).

**Table 1:** General characteristics of the assessed diets

Type of diet	Characteristics	Components
Mediterranean diet (MD) [54]	Plant-based diet originating around the Mediterranean Sea’s olive-producing regions	Olive oil as the main source of fat, cereals, vegetables, fruit, grains and legumes in abundance, seafood (frequently), moderate consumption of poultry, dairy products, nuts and alcohol, low intake of red meat, sugar, and juices
Plant-based diet [55]	Group of diets ranging from vegan to semi-vegetarian	High consumption of fiber, β-carotene, vitamin A, lutein, zeaxanthin, and vitamin C
Very low-calorie diets (VLCD) [56]	400–500 kcal per day administered in an effort to induce rapid weight loss	Meal replacement products that contain mostly proteins and carbohydrates
Low-calorie diets (LCD) [57]	1000–1300 kcal per day administered in an effort to induce slow weight loss	Consisting of approximately 20% protein, < 30% fat, and 50–60% carbohydrates of energy consumed
Gluten-free diet [58]	Excludes products containing gluten. According to the European Commission guidelines, a gluten-free product consists of less than 20 mg/kg gluten. It is the only treatment for celiac disease	Excluded products are sausages, soups, soy sauce, ice cream, wheat, rye, barley, and oats Included products are legumes, fruit, vegetables, unprocessed meat, fish, eggs, and dairy products
Low-salt diet [59]	Maximum daily sodium intake: 3 g Na or 7.5 g of sodium chloride	
Vegetarian diet [60]	Excludes meat consumption and animal slaughter by-products	Vegetable oils, nuts, soy, plant sterols, fruits, vegetables, whole grain, nuts, dairy products, honey, and eggs
Prudent diet (PD) [31]	Compared to the MD, it contains less fruits, vegetables, cereal, and fish and more red meat and juices	Root vegetables, dairy products, eggs, oil (both vegetable and olive), cereals, juices, low consumption of processed meat, fried foods, processed sweets, alcohol, and snacks
High-protein diet (HP) [61]	Almost double than recommended dietary allowance of protein	Increased protein content (1.6 g of protein/kg/day, 30% of total daily energy) (recommended protein intake 0.8 g of protein/kg/day approximately 10–15% of total daily energy)

#### Risk of Bias Assessment in Individual Studies

The risk of bias of randomized clinical trials (RCT) was assessed with the Cochrane risk of bias tool (RoB2), which assesses the following five domains: bias arising from randomization process, bias due to deviation from intervention, bias due to missing outcome data, bias in measurement of the outcome, and bias in selection of reported results. Possible risk of bias judgments was “low risk of bias,” “some concerns,” and “high risk of bias” [29]. Two reviewers (IA, AV) independently rated all RCTs included in the study for risk of bias. The tool that was used for non-randomized studies was the Cochrane risk of bias tool for non-randomized studies of intervention (ROBINS-I tool) which assesses the following 7 domains of bias: (1) confounding, (2) selection of participants into the study, (3) classification of interventions, (4) deviations from intended interventions, (5) missing data, (6) measurement of outcomes, and (7) selection of the reported result. Possible risk-of-bias judgments was “low risk of bias,” “moderate risk of bias,” “serious risk of bias,” “critical risk of bias,” and “no information” [30].

## Results

### Study Selection and Characteristics

**Fig. 1:**
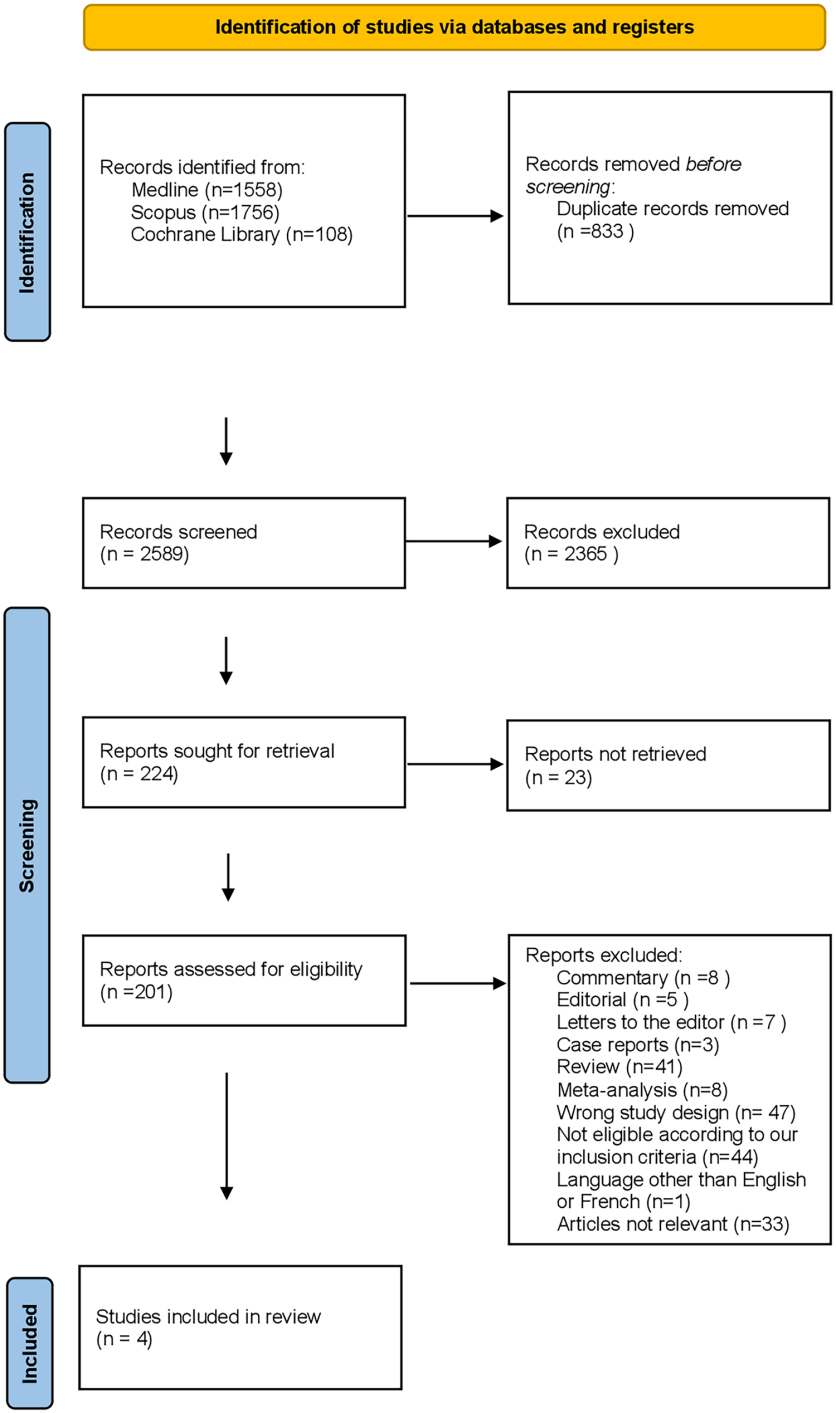
Flow diagram of study selection

The results of the study are summarized in Fig. 1. During the initial search, a total of 3422 articles were identified, of which only 201 were eligible for full-text screening. One hundred and ninety-seven articles did not meet the inclusion criteria due to wrong publication type, wrong study design, language other than English, irrelevancy, and earlier AASM scoring rules. Finally, a total of 4 articles were included in the present systematic review. Baseline characteristics of the final full-text articles are presented in Table 2.

**Table 2:** Baseline characteristics of the final full-text articles

First author/year [Ref.]	Study design	Study population	Study groups	Intervention	Outcomes/associations	Follow-up period	Results
Papandreou et al. [31]	RCT	40 OSA patients M/F: 34/6	20 assigned to the MD vs 20 assigned to a PD	MD vs PD	The primary outcome measures were the change in OSA severity measured by AHI and oxygen saturation indices and the AHI during REM sleep	6 months	Change in MD vs PD in: (1) AHI: − 15.6 ± 11.4 vs – 14 ± 22.6 (*p* = 0.791) (2) AHI/REM decrease 18.4 ± 17.6 in MD and 2.6 ± 23.7 in PD (*p* = 0.025) (3) Mean SaO_2_% in MD vs 1.5 ± 1.7 PD 1 ± 1.5 (*p* = 0.379) (4) Lowest SaO_2_% MD 4.9 ± 3.8 PD 1 ± 1.5 (*p* = 0.163) (5) Change in MedDietScore: MD 12.7 ± 4.9 PD 0.95 ± 4.5 (*p* < 0.001)
Fiori et al. [33]	RCT	56 M/F: 56/0 54 patients with severe OSA	18 assigned to placebo vs 18 assigned to sodium-restricted diet vs 18 assigned to diuretic	Placebo pill vs combination of placebo pill and sodium-restricted diet vs spironolactone plus furosemide	The primary outcome was the change in OSA severity. Secondary outcomes were lowest oxygen saturation and excessive daytime sleepiness, assessed by the Epworth Sleepiness Scale	1 week	(1) Change in AHI from baseline to follow-up was 14.4% (delta value − 7.3 events per hour, 95% confidence interval, − 13.8 to − 0.9) in the diuretic group, 22.3% (− 10.7; 95% CI, − 15.6 to − 5.7) in the diet group, and 0.8% (0.4; 95% CI, − 2.5 to 3.2) in the placebo group (*p* = 0.001 for time × group interaction) (2) Lowest oxygen saturation: placebo 77.5 ± 7.1, diet 77.9 ± 7.8, diuretic 76 ± 11.3 (3) ESS placebo 10.7 ± 5.4, diet 11.6 ± 4.8, diuretic 13.2 ± 5.3 (*p* = 0.6 for time × group interaction) (4) AHI reduction at follow-up: − 14.4% (− 13.8 to − 0.9) in the diuretic group, − 22.3% (− 10.7; 95% CI, − 15.6 to − 5.7) in the diet group, and − 0.8% (0.4; 95% CI, − 2.5 to 3.2) in the placebo group (*p* = 0.001)
Georgoulis et al. [25••]	RCT	187 OSA patients M/F: 141/46	65 assigned to SCG vs 62 assigned to MDG vs 60 assigned to MLG	Hypocaloric diet vs MD diet vs MD lifestyle	The primary outcome was participants’ AHI derived from PSG. The secondary outcomes were the proportion of patients classified as having severe OSA, the apnea index (AI), the hypopnea index (HI), and the oxygen desaturation index (ODI) as well as average and minimum SaO_2_ and parameters of sleep architecture, i.e., percent time spent in non-REM and REM sleep, sleep latency, and sleep efficiency, as well as OSA symptomatology, i.e., the degree of insomnia and sleepiness, and the proportion of patients classified as having insomnia (AIS > 6) and severe daytime sleepiness (ESS > 10)	12 months	(1) Mean AHI change − 4.2 (-7.4, -1.0) (*p* = 0.006) for the SCG, − 24.7 (− 30.4, − 19.1) (*p* < 0.001) for the MDG and − 27.3 (− 33.9, − 20.6) (*p* < 0.001) for the MLG (2) Mean ESS change − 2.34 (− 3.51, − 1.16) (*p* = 0.002) for the SCG, − 5.66 (− 7.11, − 4.22) (*p* < 0.001) for the MDG, and − 7.02 (− 8.55, − 5.49) (*p* < 0.001) for the MLG (3) Mean AIS change − 2.68 (− 3.87, − 1.49) (*p* < 0.001) for the SCG, − 5.01 (− 6.21, − 3.81) (*p* < 0.001) for the MDG, and − 4.74 (− 5.83, − 3.66) (*p* < 0.001) for the MLG (4) Mean percent body weight change: − 7.4 ± 4.1% for the MDG, − 10.6 ± 5.8% for the MLG, − 0.3 ± 3.6% for the SCG (5) Proportion of patients with excessive daytime sleepiness (SCG: 53% vs 32%, *p* = 0.002, MDG: 54% vs 5%, *p* < 0.001, MLG: 47% vs 2%, *p* < 0.001) (6) Proportion of patients with insomnia (SCG: 79% vs 60%, *p* = 0.002, MDG: 75% vs 12%, *p* < 0.001, MLG: 71% vs 14%, *p* < 0.001) (7) ODI: MDG vs SCG − 10.9 (− 20.5, − 1.35), *p* = 0.014, MLG VS SCG 12.8 (− 23.7, − 1.87) *p* = 0.001
de Melo et al. [32••]	RCT	45 OSA patients M/F: 45/0	19 assigned to low-protein group vs 18 assigned to the high-protein group	1 month	The study outcomes were the effects of a low-energy, high-protein diet on obese men’s OSA severity and metabolic parameters	1 month	(1) BMI (from 34.9 ± 4.1 to 33.7 ± 4.3 kg/m^2^ in the LP group; from 34.3 ± 3.0 to 33.1 ± 3.1 kg/m^2^ in the HP group; *p* < 0.001) (2) Fat mass (from 38.1 ± 6.1% to 37.2 ± 7.1% in the LP group; from 39.5 ± 5.5% to 38.6 ± 5.9% in the HP group; *p* = 0.009) (3) Fat-free mass (from 66.3 ± 6.5 to 63.9 ± 4.7 kg in the LP group; from 63.4 ± 6.9 to 62.4 ± 6.8 kg the in HP group; *p* < 0.001) (4) AHI (from 54.0 ± 25.0 to 33.7 ± 31.7 n/h in the LP group; from 39.7 ± 24.3 n/h to 21.4 ± 25.9 in the HP group; *p* = 0.006) (5) Arousal index (AI) (from 39.0 ± 16.4 to 24.2 ± 22.0 n/h in the LP group; from 32.2 ± 21.8 to 16.5 ± 18.5 n/h in the HP group; *p* = 0.006) (6) AI REM (from 57.0 ± 27.2 to 35.6 ± 32.6 n/h in the LP group; from 41.9 ± 23.2 to 23.9 ± 22.5 n/h in the HP group; *p* = 0.011) (7) AI NREM (from 53.8 ± 27.2 to 30.9 ± 28.9 n/h in the LP group; from 34.22 ± 22.7 to 16.7 ± 21.8 n/h in the HP group; *p* < 0.001) (8) Glucose (from 87.4 ± 40.6 to 89.5 ± 33.0 mg/dL in the LP group; from 104.3 ± 28.5 to 74.1 ± 47.7 mg/dL in the HP group; *p* < 0.05) (9) Insulin (16.9 ± 7.9 to 12.4 ± 5.7 uUI/mL in the LP group; from 16.3 ± 10.3 to 7.9 ± 6.6 uUI/mL the in HP group; *p* < 0.001) (10) Total cholesterol levels (174.0 ± 67.8 to 168.7 ± 49.1 mg/dL in the LP group; from 169.7 ± 52.0 to 134.0 ± 83.3 mg/dL in the HP group; *p* = 0.004)

*AHI* apnea hypopnea index, *AI* arousal index, *BMI* body mass index, *ESS* Epworth Sleepiness Scale, *HP* high protein, *ILI* intensive lifestyle intervention, *LP* low protein, *MD* Mediterranean diet, *MDG* Mediterranean diet group, *MLG* Mediterranean lifestyle group, *NREM* non-rapid eye movement (sleep), *OSA* obstructive sleep apnea, *PD* prudent diet, *RCT* randomized controlled trial, *REM* rapid eye movement, *SaO*_*2*_ oxygen saturation, *SCG* standard care group

### Risk of Bias Within Studies

**Fig. 2:**
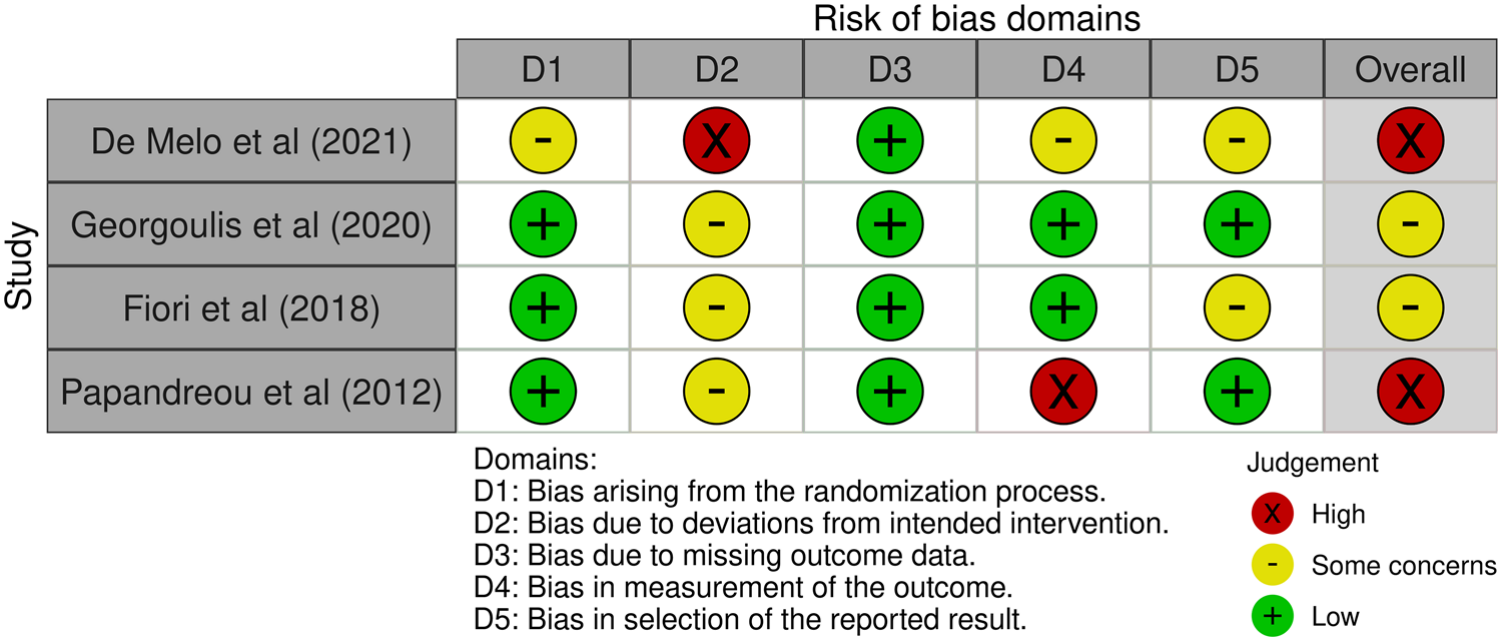
Risk-of-bias summary plot for randomized controlled trials (RoB2)

Risk of bias for RCTs was determined as “high” in two trials [31, 32••] and as “some concerns” in two trials [25••, 33]. Risk-of-bias summary plots are demonstrated in Fig. 2.

### Results of Data Synthesis

The general characteristics of the assessed diets in the included articles are described in Table 1. The present study identified 4 RCT studies of interventions for review analysis.

In the earliest study, Papandreou et al. [31] conducted a RCT to investigate the role of Mediterranean diet (MD) compared with prudent diet (PD) on severity of OSA in newly diagnosed patients for 6 months under compliant CPAP treatment. Forty participants of middle age (48.9 ± 12.7 years) with BMI ≥ 30 kg/m^2^ and moderate to severe OSA (AHI > 15/h) were included. The majority were males (85.0%) and symptomatic with an Epworth Sleepiness Scale score over 10. Participants were randomly assigned to MD (*n* = 20) and PD (*n* = 20) and received counseling for lifestyle changes and encouraged to increase their physical activity during the study follow-up. After 6 months of diet interventions, the group allocated to MD exhibited greater weight reduction (− 8.9 ± 3.9 versus − 7.2 ± 4.2 kg) although not statistically significant (*p* > 0.05). Additionally, the MD group displayed reduced AHI only during REM sleep compared with the PD group (AHI/REM dropped by 18.4 ± 17.6 in the MD vs 2.6 ± 23.7 in the PD group (*p* < 0.05) and remained significant even after adjustments for baseline AHI/REM (*p* < 0.02)). The decrease in AHI/REM was correlated with exercise scores in the International Physical Activity Questionnaire (*r* = − 0.503, *p* = 0.001) and waist circumference (*r* = 0.488, *p* = 0.002). Investigators noted that the non-significant difference in reducing neck circumference (NC) between baseline and 6 months of diet intervention could explain the lack of reduced overall AHI. This can be further clarified by the fact that NC is a stronger predictor of severity of OSA more than overall obesity [34].

In the RCT of Fiori et al. [33], the short-term effects of diuretics and salt restriction on severity of OSA and on fluid retention as a pathogenetic mechanism of OSA were assessed. The study included a total of 54 male patients aged 18–60 years with severe OSA (AHI > 30/h) and body mass index (BMI) below 35 kg/m^2^. Participants were randomized to receive (a) placebo pill (placebo group), (b) sodium-restricted diet and placebo pill (diet group), and (c) spironolactone plus furosemide (diuretic group). After 1 week of follow-up, the reduction from baseline to follow-up AHI was 14.4% (delta value − 7.3 events per hour, 95% confidence interval, − 13.8 to − 0.9) in the diuretic group, 22.3% (− 10.7; 95% CI, − 15.6 to − 5.7) in the diet group, and 0.8% (0.4; 95% CI, − 2.5 to 3.2) in the placebo group (*p* = 0.001 for time × group interaction). AHI did not return to normal values in neither of the two intervention groups. The reduction in the total body water was 2.2 ± 2.2 L in the diuretic group (*p* < 0.001) and 1.0 ± 1.6 L in the low-salt diet group (*p* = 0.002). Additionally, sleepiness as indicated by the Epworth Sleepiness Scale (ESS) significantly decreased only in the diet group, as did also neck circumference (*p* = 0.007 and *p* < 0.001 for the time × group interactions, respectively). The study demonstrated that interventions targeting at rostral fluid displacement only partially affected OSA severity.

In the MIMOSA RCT study of Georgoulis et al. [25••], 187 patients aged 49 ± 10 years with a mean BMI of 35.6 ± 6.0 kg/m^2^ and AHI of 58 [(31–85) (events/h)] were randomized to a (a) standard care group with a suggested hypocaloric diet (SCG), (b) Mediterranean diet group (MDG), and (c) Mediterranean lifestyle group (MLG). The MDG and the MLG started an intensive 6-month behavioral intervention which included seven 60-min group counseling sessions (3–5 patients), aimed at 5–10% weight loss and an increase in the level of adherence to the Mediterranean diet. In addition, CPAP was prescribed to all patients, and instructions were given for their OSA treatment. At 6 months, the mean percent body weight change was 0.3 ± 3.6% for the SCG, 7.4 ± 4.1% for the MDG, and 10.6 ± 5.8% for the MLG, significantly more for the last two groups (both *p* < 0.001). At the same period, the mean AHI (95% CI) change was − 4.2 (−7.4, − 1.0) for the SCG, − 24.7 (− 30.4, − 19.1) for the MDG, and − 27.3 (− 33.9, − 20.6) for the MLG. Although all groups exhibited statistically significant reductions in the AHI, the prevalence of severe OSA remained unchanged in the SCG (76% vs 73%, *p* = 0.63) but significantly decreased in both intervention arms (MDG: 81% vs 58%, *p* = 0.001; MLG: 69% vs 36%, *p* < 0.001). In addition, all three study groups experienced improvements in oxygenation during sleep (average and lowest SaO_2_) which were more pronounced in the two intervention arms, while the oxygen desaturation index (ODI) only decreased in intervention arms. Finally, the mean (95% CI) ESS change was − 2.34 (− 3.51, − 1.16) for the SCG, − 5.66 (− 7.11, − 4.22) for the MDG, and −7.02 (− 8.55, − 5.49) for the MLG, and the mean (95% CI) AIS change was − 2.68 (− 3.87, − 1.49) for the SCG, − 5.01 (− 6.21, − 3.81) for the MDG, and − 4.74 (− 5.83, − 3.66) for the MLG. The authors concluded that the two groups adherent to the MD diet showed significantly lower values of AHI on top of standard care, as well as a greater improvement in symptoms related to OSA; these findings were found independently of CPAP use and weight loss.

In the study of de Melo et al. [32••], consisting of 37 males with OSA, the effect of a low-protein low-energy diet (LP) compared to a high-protein low-energy (HP) over a period of 1-month follow-up was assessed. Patients were aged between 30 and 55 years and had a BMI of 34.9 ± 4.1 kg/m^2^ and 34.3 ± 3.0 kg/m^2^ in the LP and HP groups, respectively. AHI values were 54.0 ± 25.0 events/h and 39.7 ± 24.3 events/h in LP and HP groups, respectively. Following 1 month of dietary intervention for weight loss leads to a 38% reduction in AHI in the LP group and 46% in the HP group (*p* = 0.006). In addition, improvements in sleep architecture were noted in both intervention groups, namely, the arousal index (*p* = 0.006), arousal index in NREM sleep (*p* < 0.001) and in REM sleep (*p* = 0.011), and respiratory disturbance index (RDI, *p* = 0.004). Of note, RDI is the sum of AHI plus the RERAs, indicating a more detailed metric of OSA severity [28]. The study results showed an improvement in OSA symptoms and the metabolic profile, which was not statistically different between the two intervention groups, indicating the effect of weight loss due to low-energy diet, independently of the protein intake.

## Discussion

The present review addresses the effects of various types of dietary management plans on severity of OSA and OSA-related symptoms. It was found that diets such as low-salt diet and the MD can lead to mild to significant improvement of OSA severity via diet-induced weight loss. Additionally, improvement in OSA severity is feasible though a diet management plan delivered by an educational program. Moreover, diet-induced weight loss improves oxygen indices in sleep additionally to the treatment with CPAP. Finally, weight reduction and associated improvement of OSA severity via surgical intervention have better results than conservative diet weight loss; nevertheless, it carries more complications than a simple diet. Overall, all included studies have reported that diet-induced weight loss is an important aspect in the management of OSA patients, and this may result in better patient outcomes.

OSA is the most common form of sleep-disordered breathing with several detrimental effects on affected patients, mainly cardiovascular ones [1, 4]. In patients with OSA, sleep leads to decreased tone of the pharyngeal dilator muscles, reduced upper airway reflexes, loss of respiratory drive, and ultimately narrowing of the upper airway [35]. The latest evidence reports on increasing prevalence rates of OSA worldwide [5••]. This fact could be explained by increased detection rates of OSA and by increasing rates of obesity. The former results from a higher public awareness due to our understanding of OSA-associated consequences, while the latter predisposes to sleep-disordered breathing and specifically to development of apneas and hypopneas [3]. Indeed, obesity is a well-established parameter which increases the likelihood and worsens a preexisting OSA [36].

The evidence linking obesity and OSA is strong and well studied. Findings from the Wisconsin Sleep Cohort Study report that 41% of patients with OSA are overweight or obese [37]. Obese patients can develop OSA due to increased adipose tissue deposition particularly around the neck, which narrows the diameter of the upper airway. In particular, respiratory events are even worsened during sleep and in supine position due to muscle relaxation, reduced lung volumes, and loss of stimuli, all of which are present in obese patients [1]. Obesity has been also linked to dysregulation in the leptin signaling pathway, which is associated with central suppression of the upper airway neuromuscular control and development of hypoventilation [38]. Similarly, visceral fat has been found to be a mediator of inflammation affecting neuromuscular control [35, 39]. Visceral fat is more frequently present in patients with OSA than in those without OSA and is considered an important risk factor for OSA [40]. Thus, obesity and—most importantly—visceral fat deposition mediate the control of respiratory drive, destabilize breathing during sleep, and promote the development of OSA [38].

Several detrimental effects of untreated OSA are known and well characterized. The main pathophysiological event which occurs during apneas and hypopneas is intermittent hypoxia (IH) [1]. Consequently, IH gives birth to several complications such as the overactivation of the sympathetic nervous system, oxidative stress, and development or worsening of preexisting diseases [40]. When hypoxia becomes chronic, pro-inflammatory conditions emerge, and microvascular damage may occur leading to endothelial dysfunction and excess sympathetic activation [41]. This may cause insulin resistance and dyslipidemia, a potentially primary cause for hypertension and atherosclerosis [1]. Moreover, intermittent hypoxia reduces the effect of antioxidant systems during the period of hypoxia and boosts the formation of reactive oxygen species during reoxygenation leading to oxidative stress [42]. Ultimately, a strong link between cardiometabolic morbidity and OSA is formatted, setting OSA as an important risk factor for cardiovascular disease [36, 43].

Nowadays, the first-line therapy for patients with moderate to severe OSA remains the application of CPAP [43]. CPAP splints the upper airway open during sleep and protects the development of apneas and hypopneas [1]. Moreover, it prevents alveoli collapse and increases the functional residual capacity of the lungs, thereby increasing oxygenation [3]. Despite its benefits, patients often complain of nasal congestion, mouth discomfort, and claustrophobia, factors that lead to non-adherence and inadequate OSA treatment [44]. In addition, the effect of CPAP on cardiovascular outcomes is not clear and still debatable [45]. Therefore, there is an emerging need for complementary or even alternative OSA treatments on top of CPAP. To this point, current recommendations on OSA management address the role of non-CPAP options, and weight management strategies play an important role [13••, 17]. Nevertheless, further improvement is necessary, in order to implement a structured weight management plan to all patients with OSA [17]. Specifically, lifestyle interventions, e.g., diet and physical activity, are currently unpopular among most clinicians who treat patients with OSA despite the fact that they should conform to best practices for optimizing weight loss in order to have the maximum benefit for their patients [17].

Dietary programs consist of a non-invasive approach that could also mitigate the existing problems with CPAP. For example, cardiovascular outcomes can be ameliorated through plant-based diets rich in fruits and vegetables, whereas red meat (processed or not) can be more harmful [46]. Dietary management programs are an effective alternative as they can reduce the severity of OSA and improve patients’ subjective sleep complaints. Data have shown that often patients with OSA cannot tolerate higher CPAP pressures, as they feel discomfort and abandon their CPAP devices [24, 47]. Even small improvements on OSA severity through a dedicated dietary plan could improve compliance to CPAP, as lower pressures will be needed in the CPAP to splint the upper airway open [48]. Indeed, weight loss through an established diet program can synergistically act with CPAP to reduce the burden of OSA [48]. Therefore, dietary management strategies are effective in the treatment of OSA and could be initiated routinely in all patients with OSA.

Apart from diet, physical exercise may also improve severity OSA and its related cardiometabolic consequences [49], by resulting in weight loss, reducing visceral fat, and further enhancing the effects of a diet [49]. In addition, exercise may improve blood circulation, reducing fluid accumulation and its shift from the legs to the neck while reclining [49, 50]. Due to its undisputed effects, exercise can be used as an alternative treatment option for OSA patients [51, 52]. Generally, interventions that combine diet and exercise, otherwise known as lifestyle modification programs, have been proven more effective in mitigating OSA severity than diet or exercise alone [49, 53••].

### Strengths and Limitations

The present study is subject to several limitations. First, the included studies show heterogeneity, considering that different types of diet that were examined. Second, the studies have various follow-up periods ranging from 1 week to 1 year, which may differently reflect the amount of weight loss leading to incomparable results. Moreover, the search strategy of the review was not expanded to all medical libraries, and relevant articles (especially in grey literature) might also have been missed. Finally, we found a small number of studies in the literature due to our methodological criteria, but we aimed to include similar studies with the same sleep scoring criteria, as our main outcome was severity of OSA assessed by an objective sleep study using the recent criteria of sleep scoring rules [28].

Nevertheless, this review presents a few strengths. First, it includes studies that have been chosen after careful consideration of inclusion and exclusion criteria. Specifically, included were only studies where obstructive sleep apnea was evaluated using the recent definition and considering the recent sleep and respiratory scoring criteria. Therefore, the extracted data are well comparable and include a well-defined population of OSA patients. Finally, the present review reveals that weight loss through an organized diet management plan may have beneficial effects on OSA severity and shows that further research is needed to draw robust conclusions on this field.

## Conclusions

In conclusion, the present article systematically reviews the evidence on the role played by diet on OSA severity, nocturnal hypoxic parameters, and symptoms related to OSA. Mediterranean diet and low-salt diets significantly ameliorate the symptoms, but, in general, every diet leading to weight loss appears to be beneficial and improves further patients’ condition. Thus, it is important to consider this type of intervention as an additive measure in the management of OSA. However, more evidence is needed to investigate further the effect of the different diets on OSA and especially on the long-term sequelae, since the evidence is low, and the existing data is limited.

## Data Availability

All data are included in the manuscript. Additional information can be provided by the corresponding author based on reasonable request.
